# Chromosomal Arrangement of AHL-Driven Quorum Sensing Circuits in *Pseudomonas*

**DOI:** 10.5402/2012/484176

**Published:** 2012-02-29

**Authors:** Zsolt Gelencsér, Borisz Galbáts, Juan F. Gonzalez, K. Sonal Choudhary, Sanjarbek Hudaiberdiev, Vittorio Venturi, Sándor Pongor

**Affiliations:** ^1^Faculty of Information Technology, Pázmány Péter Catholic University, Práter u. 50/a, 1083 Budapest, Hungary; ^2^Bioinformatics Group, Biological Research Center, Temesvári krt 62, 6726 Szeged, Hungary; ^3^Microbiology Group, International Centre for Genetic Engineering and Biotechnology (ICGEB), Padriciano 99, 32149 Trieste, Italy; ^4^Protein Structure and Bioinformatics Group, International Centre for Genetic Engineering and Biotechnology (ICGEB), Padriciano 99, 32149 Trieste, Italy

## Abstract

*Pseudomonas* spp. are able to colonize a large variety of environments due to their wide adaptability which is also associated with an *N*-acyl homoserine lactone (AHL) gene regulation mechanism called quorum sensing (QS). In this article we present a systematic overview of the genomic arrangement patterns of quorum sensing genes found in *Pseudomonas* and compare the topologies with those found in other bacterial genomes. We find that the topological arrangement of QS genes is more variable than previously thought but there are a few unifying features that occur in many of the topological arrangements. We hypothesize that the negative regulators of QS that are often found between the canonical *luxR*/ and *luxI*-family genes may be crucial for stabilizing the output of QS circuits.

## 1. Introduction

Fluorescent *Pseudomonas* spp. are able to colonize highly dynamic environments such as soil, water, plants, as well as animals, including humans. This wide adaptability is associated with their resourceful metabolic potential and their ability to control gene expression via regulatory elements highly represented in their large genomes. For example, the opportunistic human pathogen *Pseudomonas aeruginosa* is a notorious member of this genus and is extensively studied for its ability to cause chronic human opportunistic infections in immunocompromised patients [1]. In addition to human pathogens, also important plant pathogens are present in this group of bacteria*; Pseudomonas syringae* is an important model of plant pathogenic bacteria since its pathovars can infect many different plants (c.f. http://www.pseudomonas-syringae.org/). Plant-growth-promoting fluorescent pseudomonads are also studied for their ability to colonize plant-related niches, like the rhizosphere (e.g., *P. fluorescens*, *P. Putida,* and *P. chlororaphis*), where they can act as plant beneficial bacteria either by antagonizing plant deleterious microorganisms or by directly influencing plant disease resistance and growth [2].

Bacteria often possess a regulatory system, known as quorum sensing (QS), to modulate gene expression as a function of their cell density (for reviews see [3, 4]). In Gram negative bacteria, the most common QS system is regulated by the *N*-acyl homoserine lactone signaling molecules (AHLs). Studies of the mechanisms and role of QS in several *Pseudomonas* spp. indicated that the most common signal molecules used are in fact AHLs [5]. These signals were first described in the marine bioluminescent bacterium *Vibrio fischeri* in which QS regulates light production (reviewed by [6]). The model *N*-AHL QS system consists of two proteins belonging to the LuxI and LuxR families, respectively, [4, 7]. LuxI-family proteins are cytoplasmic enzymes responsible for AHL synthesis [8]. AHLs are synthesized from S-adenosyl methionine, which provides the homoserine lactone moiety, and acyl carrier protein, which provides the fatty acyl moiety. After synthesis, the signal can move freely across the bacterial membranes and accumulates both intra- and extracellularly in proportion to cell density. Above a critical threshold concentration or cell density, AHLs interact directly with the LuxR-family protein, which in most cases results in the formation of homodimers. These complexes can then bind at specific sequences called *lux*-boxes that are located in the promoter region of target QS-regulated genes, affecting their expression.

Many important phenotypes are regulated by AHL QS and QS has been suggested as a possible target to control bacterial colonization [9]. QS regulates phenotypes related to both pathogenesis (virulence associated factors like toxins, motility, secreted enzymes, and biofilm-related genes/proteins) and to beneficial effects in plant growth promoting rhizobacteria (PGPR) (e.g., production of antibiotic and antifungal compounds and induction of systemic resistance in the plant [10–13]).

AHL QS is particularly interesting in the pseudomonads due to the presence, diversity, and complexity of regulatory circuits present in various species. In the case of *P. aeruginosa,* AHL QS seems conserved and ubiquitous, being composed of a complex hierarchy of two LuxI/R pairs and a series of regulators [5, 14]. In fact, it has been estimated that quorum sensing regulates up to 3% of *P. aeruginosa* genes. On the other hand, most strains of *P. fluorescens* and *P. putida* do not possess an AHL QS system [15, 16]. In this study, we performed an in-depth systematic study on the chromosomal arrangement and synteny of AHL QS systems in pseudomonads in order to determine the commonalities and differences that may exist between pseudomonads and other bacteria. Previous studies concentrated either on the presence or absence of AHL QS genes in bacteria [17], or on the regulatory design principles of selected QS systems [18, 19]. Here, we present a survey of AHL-driven QS circuits in pseudomonads and compare the chromosomal arrangements with those found in other bacterial genomes.

## 2. QS Genes in Complete Bacterial Genomes

We used the sequence data of 1346 full bacterial proteomes found at the NCBI bacterial genome repository as well as published QS operon sequences from NCBI GenBank (data last accessed on June 12, 2011). Draft genome sequences were excluded from the analysis because of the uncertain annotations we found in some of them. The search included standard bioinformatics methodologies and manual curation (see supplementary materials available online at doi: 10.5402/2012/484176). We started our search for *luxR* sensor/regulators, *luxI* AHL synthases, *rsaL,* and *rsaM* repressor homologues in complete bacterial genomes and included only a set of selected examples of *Pseudomonas* data from incomplete genomes (Tables 1 and 2). For *luxR*, *luxI*, *rsaL, *and *rsaM* we use the symbols *R*, *I, L,* and *M*, respectively, and refer to them as “QS genes”. Solo *R* genes [20] as well as other lonely occurrences of QS genes were not considered. This cautious approach of manual curation was adopted because we were primarily interested in the genomic arrangements and not so much in finding hitherto unannotated genes in the complete genomes. Still we found a few unannotated genes that were accepted on the condition that they were in one of the previously observed topological arrangements. From a total of over 4.3 million genes analyzed, we found 624 *R* genes (29 unannotated), 269 *I* genes (12 unannotated), 39 *L* genes (11 unannotated), and 36 *M* genes (36 unannotated). Out of the 1346 complete genomes, 143 were found to contain QS genes in the vicinity of other QS genes (i.e., within a distance of 3000 nt). All of these were proteobacterial genomes. We do not consider our analysis as comprehensive because, among other things, it was based on the reading frames given in the genome annotations, and we left *Rhizobia* and *Agrobacterium* species out of the survey because the arrangement of their QS genes is different from *Pseudomonas*. We found a few conflicts with respect to the gene functions assigned in the genome annotations but not in *Pseudomonas*.

## 3. Genomic Topologies of AHL-Driven QS Circuits

We found two major types of topological arrangements that we term *RI* and *RXI*, respectively, (Tables 1 and 2). In RI, the two genes are vicinal while in *RXI* there is at least one additional gene between the two *LuxI* and *LuxR* family genes.

### 3.1. The RI Topology

There are 3 possible variations, namely tandem (unidirectional), convergent, divergent. All of these are found in proteobacteria, *Pseudomonas* does not seem to contain the divergent topology which can, however, be found in other gamma proteobacteria.

### 3.2. The RXI Topology

In these topologies, one or more genes are found between the *R* and the *I* genes. All the 8 possible arrangements are found in proteobacteria, however, only 3 in *Pseudomonas* where the *X* gene is most frequently *L* (RsaL, found in *P. aeruginosa*, *P. putida,* and *P. fuscovaginae* species). *M* is much more frequently found in *Burkholderia*, the only *Pseudomonas* to contain *M* is *P. fuscovaginae*, which is at the same time, the only pseudomonad found so far to contain both *L* and *M* genes. Both the *L* and the *M* genes have their canonical topologies which are shown in the table separately, denoted as RLI (*L*1) and *RMI* (*M*1), respectively. Both of these topologies can be found both in *Burkholderia* and in *Pseudomonas*. However, some *Burkholderia* species contain an additional copy of *M*, which is in a non-canonical arrangement, either because there are one to five additional genes betweem *R* and *M* (*M*2 topology, found in *B. pseudomallei* 1106a, *B. pseudomallei* 1710b, *B. pseudomallei* 668, *B. pseudomallei* K96243, *B. thailandensis* E264), or because the otherwise constitutive *R* gene is missing in the immediate vicinity of the MI tandem (*M*3 topology, *B. ambipharia* AMMD, and MC40-6). On the other hand, *P. fluorescens* NCIMB 10586 contains a gene coding for an enzyme, *mupX* in the X position [21].

## 4. The X Genes

In *Pseudomonas*, the genes in the X position are predominantly negative regulators of the QS response. *RsaL* (*L*) [22] was shown to belong to the tetrahelical superclass of H-T-H proteins [23]. Members of this family are widespread repressors in bacteria and bind to DNA as dimers. We found that homologues of *RsaL* frequently occur outside QS circuits in various bacterial genomes (data not shown). In *P. fuscovaginae, RsaL* binds to DNA next to the lux box and prevents expression of the *R* gene [24]. In contrast, *RsaM* (*M*) is a protein of unknown structure that seems to occur only in the context of QS circuits. *M* was found to negatively regulate QS in *P. fuscovaginae* [24]. Finally, *mupX* of *P. fluorescens* NCIMB10586 is an amidase-hydrolase that was shown to degrade the AHL signal produced by the same species thereby decreasing the QS response [21].

## 5. Overlapping Genes

 Two topologies, R2 and L1, contain overlaps at the proximal ends of convergent genes. Such overlaps are not uncommon in tightly coregulated gene circuits of bacteria [25], for instance restriction modification systems [26]. The R2 type of arrangements in *P. syringae* contain overlapping *R* and *I* genes (2-to 68 nt) while *P. fluorescens* genomes do not. In the *L*1 type QS circuits of *P. aeruginosa,* the overlaps are 10 nt while in *P. fuscovaginae* the overlap is 20 nt. On the contrary, the *L*1 circuits of *P. putida* are not overlapping, though the open reading frames of *R* and *L* are only *4* nt apart. Tsai and Winans noted that the overlapping *R*2-like arrangement is common to QS circuits in which *R* proteins are able to fold, dimerize, bind DNA, and regulate transcription in the absence of AHLs; moreover, these proteins are antagonized by their cognate AHLs [27]. The same authors also argued that the expression of one member of a convergent and overlapping gene pair might be antagonized by the expression of the other member, either via *RNA* polymerase collisions or by hybridization of the two complementary *mRNA*s [27].

## 6. Regulatory Implications

The most conspicuous feature of the various circuit topologies is the potential negative regulatory effect of *R* on *I* which, as mentioned above, goes in parallel with the well known positive regulatory effect. In other words, *R* seems both to activate and to inhibit the *I* genes in a number of cases. In *RXI* circuits, *R* activates an *X* gene that decreases the effect of *I*. In the *R*2 circuits, the negative effect follows from the overlap between the convergently transcribed *R* and *I* genes [27]. Regulatory circuits in which an element can both activate and inhibit another element are termed incoherent feed forward loops or *IFFL*s [41, 42]. In contrast to simple feed forward arrangements, *IFFL*s can exhibit a number of complex behavior patterns (for a review see [43]). Perhaps the most important of these is the stabilization of the output signals: while simple feed forward circuits have no inherent limits on their output, IFFL networks have bounded output which ensures robustness against fluctuations in the input signal levels. Most often, QS regulatory circuits are simply referred to as autoinduction loops which, at least in theory, should increase their output without limits. The examples shown in this survey suggest that a stabilizing, negative regulatory pathway is present in many QS systems. It was found experimentally that deletion of *RsaL* or *RsaM* leads to a dramatic increase in AHL production, but the resulting mutants are less virulent than the wild type [24], which shows, on the other hand, that the negative regulatory path may in fact be a crucial stabilizing element within the QS circuits.

Finally, we mention that the chromosomal arrangements found in QS genes seem more varied than expected so the search for common regulatory principles remains an important task for future research.

## Supplementary Material

List of protein sequences, genomes and description of bioinformatics tools.

## Figures and Tables

**Table 1:** Typical chromosomal arrangements of AHL-driven quorum sensing circuits in *Pseudomonas*.

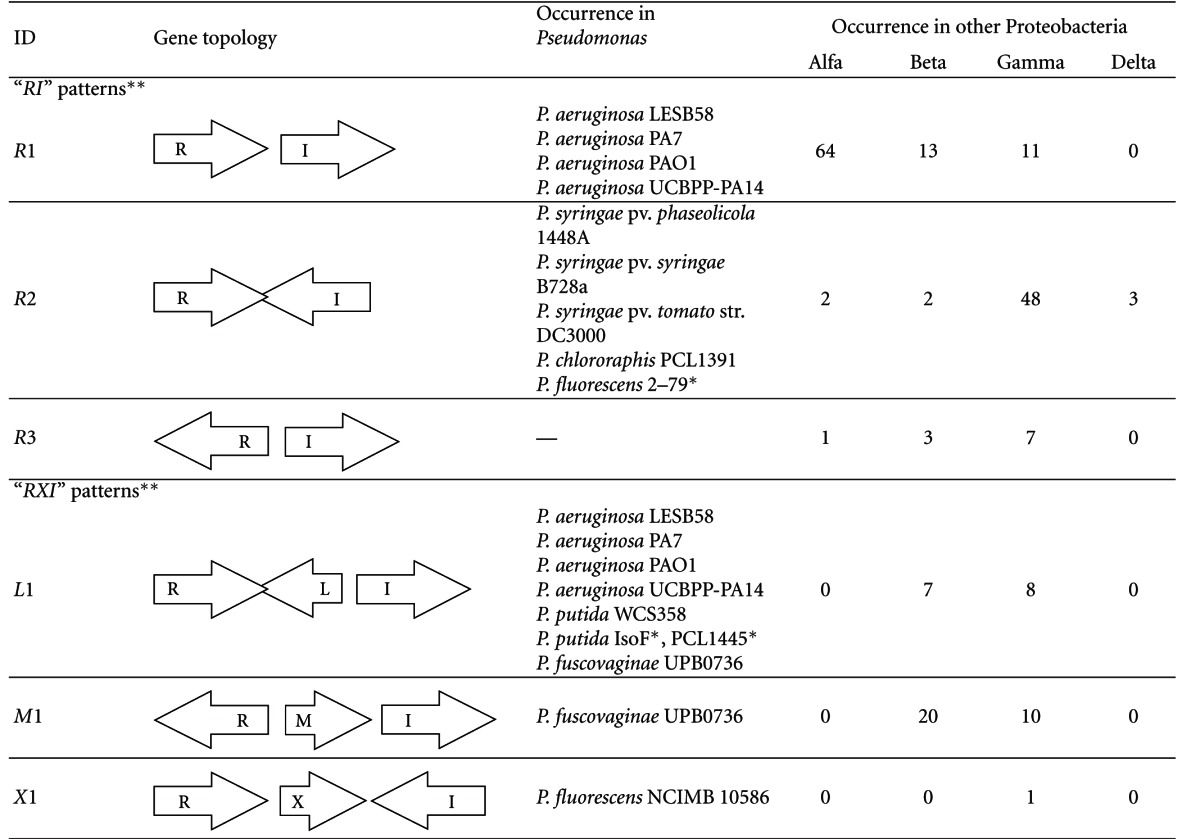

*Pattern does not contain the overlap indicated.

**RI patterns = two genes are in vicinity and *RXI* patterns = one additional gene is between luxR and LuxI.

**Table 2:** Examples of *Pseudomonas* species with of AHL-driven quorum sensing networks.

*Pseudomonas* species	QS circuit	Pattern id (from Table 1)
*P*. *aeruginosa* LESB58 [28]	rhlR/rhlI	R1
	lasR/rsaL/lasI	L1
*P. aeruginosa* PA7 [29]	rhlR/rhlI	R1
	lasR/rsaL/lasI	L1
*P. aeruginosa* PAO1 [30]	rhlR/rhlI	R1
	lasR/rsaL/lasI	L1
*P. aeruginosa* UCBPP-PA14 [31]	rhlR/rhlI	R1
	lasR/rsaL/lasI	L1
*P. fuscovaginae* UPB0736 [24]	sR/rsaM/sI	M1
	vR/rsaL/vI	L1
*P. syringae* pv. *phaseolicola* 1448A [32]	AhlR/AhlI	R2
*P. syringae* pv. *syringae* B728a [33]	Psyr_1622/Psyr_1621	R2
*P. syringae* pv. *tomato* str. DC3000 [34]	psyR/psyI	R2
*P. chlororaphis* PCL1391 [35, 36]	phzR/PhzI	R2
*P. fluorescens* 2–79 [37]	phzR/PhzI	R2
*P. fluorescens* NCIMB 10586 [38]	mupR/mupX/mupI	X1
*P. putida* WCS358 [39]	uR/rsaL/uI	L1
*P. putida* IsoF [40]	ppuR/rsaL/ppuI	L1
*P. putida* PCL1445 [11]	ppuR/rsaL/ppuI	L1
