# Supplementary material for: Chromosomal Arrangement of AHL-Driven Quorum Sensing Circuits in Pseudomonas
Source: ISRN Microbiol. 2012 Feb 29;2012:484176. doi: 10.5402/2012/484176 (PMC3658600; doi:10.5402/2012/484176)
Supplement: Supplementary file 1 — List of protein sequences, genomes and description of bioinformatics tools. [file 484176.f1.pdf]

Supplementary materials
Zs. Gelencsér et al: Chromosomal arrangement of AHL-driven quorum sensing circuits in *Pseudomonas*

The distribution and arrangement of *LuxR*, *LuxI*, *rsaL* and *rsaM* gene homologues was surveyed in the Genome and GenBank sections of the NCBI (http://ncbi.nlm.nih.gov/) and the UNIPROT database (http:/www.uniprot.org/). The analysis was based on a core set of proteins for the 4 groups, listed below:

Core protein sets:

LuxR homologues:

Mesorhizobium loti MAFF303099	NP_106261.1
Mesorhizobium loti MAFF303099	NP_106660.1
Mesorhizobium loti MAFF303099	NP_109411.1
Pseudomonas aeruginosa PAO1	NP_250121.1
Pseudomonas aeruginosa PAO1	NP_252167.1
Sinorhizobium meliloti 1021	NP_385944.1
Ralstonia solanacearum GMI1000	NP_521406.1
Ralstonia solanacearum GMI1000	NP_522339.1
Yersinia pestis KIM 10	NP_669049.1
Yersinia pestis KIM 10	NP_670674.1
Bradyrhizobium japonicum USDA 110	NP_767702.1
Pseudomonas syringae pv. tomato str. DC3000	NP_793635.1
Chromobacterium violaceum ATCC 12472	NP_903760.1
Rhodopseudomonas palustris CGA009	NP_945674.1
Yersinia pestis biovar Microtus str. 91001	NP_993605.1
Yersinia pestis biovar Microtus str. 91001	NP_994736.1
Pectobacterium atrosepticum SCRI1043	YP_048234.1
Yersinia pseudotuberculosis IP 32953	YP_071012.1
Yersinia pseudotuberculosis IP 32953	YP_071752.1
Burkholderia mallei ATCC 23344	YP_106160.1
Burkholderia mallei ATCC 23344	YP_105961.1
Burkholderia pseudomallei K96243	YP_110896.1
Burkholderia pseudomallei K96243	YP_111575.1
Ruegeria pomeroyi DSS-3	YP_165634.1
Ruegeria pomeroyi DSS-3	YP_167510.1
Vibrio fischeri ES114	YP_206883.1
Pseudomonas syringae pv. syringae B728a	YP_234708.1
Pseudomonas syringae pv. phaseolicola 1448A	YP_273861.1

Nitrobacter winogradskyi Nb-255	YP_317246.1
Burkholderia pseudomallei 1710b	YP_335776.1
Burkholderia pseudomallei 1710b	YP_337635.1
Burkholderia sp. 383	YP_371810.1
Rhodospirillum rubrum ATCC 11170	YP_428476.1
Burkholderia thailandensis E264	YP_439002.1
Burkholderia thailandensis E264	YP_439706.1
Sodalis glossinidius str. 'morsitans'	YP_453965.1
Rhizobium etli CFN 42	YP_470410.1
Rhizobium etli CFN 42	YP_473056.1
Rhodopseudomonas palustris HaA2	YP_484040.1
Rhodopseudomonas palustris HaA2	YP_486928.1
Jannaschia sp. CCS1	YP_508561.1
Saccharophagus degradans 2-40	YP_528967.1
Rhodopseudomonas palustris BisB18	YP_530593.1
Rhodopseudomonas palustris BisB18	YP_531902.1
Burkholderia xenovorans LB400	YP_554691.1
Burkholderia xenovorans LB400	YP_555670.1
Rhodopseudomonas palustris BisB5	YP_567541.1
Rhodopseudomonas palustris BisB5	YP_569310.1
Sphingopyxis alaskensis RB2256	YP_617565.1
Sphingopyxis alaskensis RB2256	YP_617627.1
Burkholderia cenocepacia AU 1054	YP_623508.1
Yersinia pestis Antiqua	YP_650193.1
Yersinia pestis Antiqua	YP_651866.1
Yersinia pestis Nepal516	YP_647982.1
Yersinia pestis Nepal516	YP_649110.1
Pseudoalteromonas atlantica T6c	YP_659944.1
Chelativorans sp. BNC1	YP_674864.1
Roseobacter denitrificans OCh 114	YP_681951.1
Burkholderia ambifaria AMMD	YP_776003.1
Rhodopseudomonas palustris BisA53	YP_781245.1
Pseudomonas aeruginosa UCBPP-PA14	YP_791822.1
Pseudomonas aeruginosa UCBPP-PA14	YP_789670.1
Rhizobium leguminosarum bv. viciae 3841	YP_768957.1
Burkholderia cenocepacia HI2424	YP_838351.1
Aeromonas hydrophila subsp. hydrophila ATCC 7966	YP_855090.1
Paracoccus denitrificans PD1222	YP_914594.1
Acidovorax citrulli AAC00-1	YP_972129.1
Burkholderia mallei SAVP1	YP_989940.1
Yersinia enterocolitica subsp. enterocolitica 8081	YP_001005891.1

Burkholderia mallei NCTC 10229	YP_001024423.1
Burkholderia mallei NCTC 10229	YP_001025820.1
Burkholderia pseudomallei 668	YP_001062292.1
Burkholderia pseudomallei 668	YP_001063209.1
Burkholderia mallei NCTC 10247	YP_001078154.1
Burkholderia mallei NCTC 10247	YP_001077903.1
Burkholderia pseudomallei 1106a	YP_001076161.1
Burkholderia pseudomallei 1106a	YP_001075258.1
Acinetobacter baumannii ATCC 17978	YP_001083200.1
Burkholderia vietnamiensis G4	YP_001114942.1
Burkholderia vietnamiensis G4	YP_001117674.1
Aeromonas salmonicida subsp. salmonicida A449	YP_001143472.1
Yersinia pestis Pestoides F	YP_001161917.1
Yersinia pestis Pestoides F	YP_001163230.1
Bradyrhizobium sp. ORS 278	YP_001203095.1
Bradyrhizobium sp. BTAi1	YP_001220570.1
Bradyrhizobium sp. BTAi1	YP_001241092.1
Bradyrhizobium sp. BTAi1	YP_001242900.1
Geobacter uraniireducens Rf4	YP_001231850.1
Sinorhizobium medicae WSM419	YP_001327236.1
Pseudomonas aeruginosa PA7	YP_001349253.1
Pseudomonas aeruginosa PA7	YP_001347033.1
Yersinia pseudotuberculosis IP 31758	YP_001399708.1
Yersinia pseudotuberculosis IP 31758	YP_001400524.1
Serratia proteamaculans 568	YP_001476304.1
Dinoroseobacter shibae DFL 12	YP_001531661.1
Dinoroseobacter shibae DFL 12	YP_001534186.1
Burkholderia multivorans ATCC 17616	YP_001583946.1
Gluconacetobacter diazotrophicus PAl 5	YP_001603072.1
Yersinia pestis Angola	YP_001606210.1
Yersinia pestis Angola	YP_001604810.1
Methylobacterium extorquens PA1	YP_001641953.1
Acinetobacter baumannii AYE	YP_001715477.1
Yersinia pseudotuberculosis YPIII	YP_001719545.1
Yersinia pseudotuberculosis YPIII	YP_001720401.1
Shewanella woodyi ATCC 51908	YP_001761363.1
Burkholderia cenocepacia MC0-3	YP_001777917.1
Burkholderia cenocepacia MC0-3	YP_001779191.1
Methylobacterium sp. 4-46	YP_001772212.1
Methylobacterium radiotolerans JCM 2831	YP_001776815.1
Methylobacterium radiotolerans JCM 2831	YP_001758389.1

Methylobacterium radiotolerans JCM 2831	YP_001783296.1
Burkholderia ambifaria MC40-6	YP_001811253.1
Beijerinckia indica subsp. indica ATCC 9039	YP_001832058.1
Acinetobacter baumannii ACICU	YP_001844797.1
Burkholderia phymatum STM815	YP_001860599.1
Yersinia pseudotuberculosis PB1/+	YP_001873010.1
Yersinia pseudotuberculosis PB1/+	YP_001873807.1
Burkholderia phytofirmans PsJN	YP_001888024.1
Burkholderia phytofirmans PsJN	YP_001893790.1
Erwinia tasmaniensis Et1/99	YP_001906896.1
Erwinia tasmaniensis Et1/99	YP_001908006.1
Methylobacterium populi BJ001	YP_001927660.1
Burkholderia multivorans ATCC 17616	YP_001948918.1
Rhizobium etli CIAT 652	YP_001979199.1
Rhizobium etli CIAT 652	YP_001985289.1
Rhodopseudomonas palustris TIE-1	YP_001989359.1
Rhodopseudomonas palustris TIE-1	YP_001991323.1
Phenylobacterium zucineum HLK1	YP_002128523.1
Vibrio fischeri MJ11	YP_002158591.1
Acidithiobacillus ferrooxidans ATCC 53993	YP_002220093.1
Burkholderia cenocepacia J2315	YP_002232873.1
Burkholderia cenocepacia J2315	YP_002234479.1
Rhizobium leguminosarum bv. trifolii WSM2304	YP_002282164.1
Aliivibrio salmonicida LFI1238	YP_002265247.1
Acinetobacter baumannii AB0057	YP_002317567.1
Acinetobacter baumannii AB307-0294	YP_002327279.1
Methylocella silvestris BL2	YP_002360441.1
Methylobacterium chloromethanicum CM4	YP_002423670.1
Acidithiobacillus ferrooxidans ATCC 23270	YP_002426403.1
Pseudomonas aeruginosa LESB58	YP_002439139.1
Pseudomonas aeruginosa LESB58	YP_002441567.1
Yersinia pestis CO92	YP_002346032.1
Yersinia pestis CO92	YP_002347421.1
Methylobacterium nodulans ORS 2060	YP_002495629.1
Methylobacterium nodulans ORS 2060	YP_002496262.1
Methylobacterium nodulans ORS 2060	YP_002497059.1
Geobacter sp. FRC-32	YP_002537872.1
Agrobacterium radiobacter K84	YP_002541325.1
Agrobacterium vitis S4	YP_002551488.1
Agrobacterium vitis S4	YP_002549361.1
Sinorhizobium fredii NGR234	YP_002826207.1

Burkholderia glumae BGR1	YP_002909041.1
Candidatus Hamiltonella defensa 5AT (Acyrthosiphon pisum)	YP_002923741.1
Edwardsiella ictaluri 93-146	YP_002934275.1
Variovorax paradoxus S110	YP_002947664.1
Desulfovibrio magneticus RS-1	YP_002955225.1
Methylobacterium extorquens AM1	YP_002965846.1
Methylobacterium extorquens AM1	YP_002966880.1
Rhizobium leguminosarum bv. trifolii WSM1325	YP_002976727.1
Dickeya zeae Ech1591	YP_003002472.1
Pectobacterium carotovorum subsp. carotovorum PC1	YP_003019697.1
Methylobacterium extorquens DM4	YP_003070967.1
Erwinia pyrifoliae Ep1/96	YP_002649216.1
Pectobacterium wasabiae WPP163	YP_003261727.1
Halothiobacillus neapolitanus c2	YP_003262848.1
Edwardsiella tarda EIB202	YP_003296639.1
Dickeya dadantii Ech586	YP_003331714.1
Citrobacter rodentium ICC168	YP_003366469.1
Pantoea ananatis LMG 20103	YP_003520251.1
Erwinia amylovora CFBP1430	YP_003530769.1
Erwinia amylovora ATCC 49946	YP_003538485.1
Sphingobium japonicum UT26S	YP_003546444.1
Shewanella violacea DSS12	YP_003558208.1
Yersinia pestis Z176003	YP_003566925.1
Yersinia pestis Z176003	YP_003568279.1
Rhodobacter capsulatus SB 1003	YP_003576500.1
Burkholderia sp. CCGE1002	YP_003608086.1
Pantoea vagans C9-1	YP_003729882.1
Acinetobacter sp. DR1	YP_003734010.1
Ralstonia solanacearum PSI07	YP_003750859.1
Ralstonia solanacearum PSI07	YP_003749681.1
Ralstonia solanacearum CFBP2957	YP_003744152.1
Erwinia billingiae Eb661	YP_003740504.1
Erwinia billingiae Eb661	YP_003740953.1
Gallionella capsiferriformans ES-2	YP_003847232.1
Dickeya dadantii 3937	YP_003885142.1
Burkholderia sp. CCGE1003	YP_003910271.1
Pantoea vagans C9-1	YP_003930459.1
Ketogulonicigenium vulgare Y25	YP_003964947.1
Enterobacter cloacae SCF1	YP_003941575.1
Rhodomicrobium vannielii ATCC 17100	YP_004012994.1
Asticcacaulis excentricus CB 48	YP_004088229.1

Rhodopseudomonas palustris DX-1	YP_004106680.1
Rhodopseudomonas palustris DX-1	YP_004106955.1
Rhodopseudomonas palustris DX-1	YP_004108424.1
Pantoea sp. At-9b	YP_004115278.1
Mesorhizobium ciceri biovar biserrulae WSM1271	YP_004144717.1
Mesorhizobium ciceri biovar biserrulae WSM1271	YP_004145052.1
Burkholderia sp. CCGE1001	YP_004230807.1

LuxI homologues

Mesorhizobium loti MAFF303099	NP_106262.1
Mesorhizobium loti MAFF303099	NP_106661.1
Mesorhizobium loti MAFF303099	NP_109412.1
Pseudomonas aeruginosa PAO1	NP_250123.1
Pseudomonas aeruginosa PAO1	NP_252166.1
Sinorhizobium meliloti 1021	NP_385945.1
Ralstonia solanacearum GMI1000	NP_521405.1
Ralstonia solanacearum GMI1000	NP_522340.1
Yersinia pestis KIM 10	NP_669050.1
Yersinia pestis KIM 10	NP_670673.1
Bradyrhizobium japonicum USDA 110	NP_767703.1
Pseudomonas syringae pv. tomato str. DC3000	NP_793636.1
Chromobacterium violaceum ATCC 12472	NP_903761.1
Rhodopseudomonas palustris CGA009	NP_945673.1
Yersinia pestis biovar Microtus str. 91001	NP_993604.1
Yersinia pestis biovar Microtus str. 91001	NP_994737.1
Pectobacterium atrosepticum SCRI1043	YP_048233.1
Yersinia pseudotuberculosis IP 32953	YP_071011.1
Yersinia pseudotuberculosis IP 32953	YP_071751.1
Burkholderia mallei ATCC 23344	YP_106161.1
Burkholderia mallei ATCC 23344	YP_105963.1
Burkholderia pseudomallei K96243	YP_110894.1
Burkholderia pseudomallei K96243	YP_111576.1
Ruegeria pomeroyi DSS-3	YP_165635.1
Ruegeria pomeroyi DSS-3	YP_167511.1
3-oxo-C6-HSL autoinducer synthesis protein LuxI [Vibrio fischeri ES114	YP_206882.1
Pseudomonas syringae pv. syringae B728a	YP_234707.1
Pseudomonas syringae pv. phaseolicola 1448A	YP_273860.1
Nitrobacter winogradskyi Nb-255	YP_317245.1
Burkholderia pseudomallei 1710b	YP_337633.1
Burkholderia pseudomallei 1710b	YP_335777.1

Burkholderia sp. 383	YP_371808.1
Rhodospirillum rubrum ATCC 11170	YP_428477.1
Burkholderia thailandensis E264	YP_439708.1
Burkholderia thailandensis E264	YP_439001.1
Sodalis glossinidius str. 'morsitans'	YP_453964.1
Rhizobium etli CFN 42	YP_470411.1
Rhizobium etli CFN 42	YP_473057.1
Rhodopseudomonas palustris HaA2	YP_484039.1
Rhodopseudomonas palustris HaA2	YP_486927.1
Jannaschia sp. CCS1	YP_508562.1
Saccharophagus degradans 2-40	YP_528965.1
Rhodopseudomonas palustris BisB18	YP_530592.1
Rhodopseudomonas palustris BisB18	YP_531903.1
Burkholderia xenovorans LB400	YP_554693.1
Burkholderia xenovorans LB400	YP_555669.1
Rhodopseudomonas palustris BisB5	YP_567542.1
Rhodopseudomonas palustris BisB5	YP_569311.1
Sphingopyxis alaskensis RB2256	YP_617566.1
Sphingopyxis alaskensis RB2256	YP_617628.1
Burkholderia cenocepacia AU 1054	YP_623506.1
Yersinia pestis Antiqua	YP_650194.1
Yersinia pestis Antiqua	YP_651865.1
Yersinia pestis Nepal516	YP_647981.1
Yersinia pestis Nepal516	YP_649109.1
Pseudoalteromonas atlantica T6c	YP_659946.1
Chelativorans sp. BNC1	YP_674865.1
Roseobacter denitrificans OCh 114	YP_681952.1
Burkholderia ambifaria AMMD	YP_776005.1
Rhodopseudomonas palustris BisA53	YP_781244.1
Pseudomonas aeruginosa UCBPP-PA14	YP_791820.1
Pseudomonas aeruginosa UCBPP-PA14	YP_789671.1
Rhizobium leguminosarum bv. viciae 3841	YP_768958.1
Burkholderia cenocepacia HI2424	YP_838353.1
Aeromonas hydrophila subsp. hydrophila ATCC 7966	YP_855089.1
Paracoccus denitrificans PD1222	YP_914595.1
Acidovorax citrulli AAC00-1	YP_972130.1
Burkholderia mallei SAVP1	YP_989942.1
Yersinia enterocolitica subsp. enterocolitica 8081	YP_001005892.1
Burkholderia mallei NCTC 10229	YP_001024425.1
Burkholderia mallei NCTC 10229	YP_001025818.1
Burkholderia pseudomallei 668	YP_001063210.1

Burkholderia pseudomallei 668	YP_001062290.1
Burkholderia mallei NCTC 10247	YP_001077901.1
Burkholderia mallei NCTC 10247	YP_001078152.1
Burkholderia pseudomallei 1106a	YP_001076162.1
Burkholderia pseudomallei 1106a	YP_001075256.1
Acinetobacter baumannii ATCC 17978	YP_001083198.1
Burkholderia vietnamiensis G4	YP_001114940.1
Burkholderia vietnamiensis G4	YP_001117676.1
Aeromonas salmonicida subsp. salmonicida A449	YP_001143471.1
Yersinia pestis Pestoides F	YP_001161918.1
Yersinia pestis Pestoides F	YP_001163229.1
Bradyrhizobium sp. ORS 278	YP_001203094.1
Bradyrhizobium sp. BTAi1	YP_001220569.1
Bradyrhizobium sp. BTAi1	YP_001241094.1
Bradyrhizobium sp. BTAi1	YP_001242901.1
Geobacter uraniireducens Rf4	YP_001231849.1
Sinorhizobium medicae WSM419	YP_001327237.1
Pseudomonas aeruginosa PA7	YP_001347034.1
Pseudomonas aeruginosa PA7	YP_001349251.1
Yersinia pseudotuberculosis IP 31758	YP_001399709.1
Yersinia pseudotuberculosis IP 31758	YP_001400525.1
Serratia proteamaculans 568	YP_001476305.1
Dinoroseobacter shibae DFL 12	YP_001531662.1
Dinoroseobacter shibae DFL 12	YP_001534185.1
Burkholderia multivorans ATCC 17616	YP_001583944.1
Gluconacetobacter diazotrophicus PAl 5	YP_001603070.1
Yersinia pestis Angola	YP_001604809.1
Yersinia pestis Angola	YP_001606209.1
Methylobacterium extorquens PA1	YP_001641952.1
Acinetobacter baumannii AYE	YP_001715479.1
Yersinia pseudotuberculosis YPIII	YP_001719546.1
Yersinia pseudotuberculosis YPIII	YP_001720402.1
Shewanella woodyi ATCC 51908	YP_001761364.1
Burkholderia cenocepacia MC0-3	YP_001777918.1
Burkholderia cenocepacia MC0-3	YP_001779189.1
Methylobacterium sp. 4-46	YP_001772211.1
Methylobacterium radiotolerans JCM 2831	YP_001776814.1
Methylobacterium radiotolerans JCM 2831	YP_001758390.1
Methylobacterium radiotolerans JCM 2831	YP_001783295.1
Burkholderia ambifaria MC40-6	YP_001811255.1
Beijerinckia indica subsp. indica ATCC 9039	YP_001832057.1

Acinetobacter baumannii ACICU	YP_001844795.1
Burkholderia phymatum STM815	YP_001860597.1
Yersinia pseudotuberculosis PB1/+	YP_001873009.1
Yersinia pseudotuberculosis PB1/+	YP_001873806.1
Burkholderia phytofirmans PsJN	YP_001888022.1
Burkholderia phytofirmans PsJN	YP_001893789.1
Erwinia tasmaniensis Et1/99	YP_001906897.1
Erwinia tasmaniensis Et1/99	YP_001908005.1
Methylobacterium populi BJ001	YP_001927659.1
Burkholderia multivorans ATCC 17616	YP_001948920.1
Rhizobium etli CIAT 652	YP_001979200.1
Rhizobium etli CIAT 652	YP_001985290.1
Rhodopseudomonas palustris TIE-1	YP_001989358.1
Rhodopseudomonas palustris TIE-1	YP_001991324.1
Phenylobacterium zucineum HLK1	YP_002128524.1
Vibrio fischeri MJ11	YP_002158590.1
Acidithiobacillus ferrooxidans ATCC 53993	YP_002220095.1
Burkholderia cenocepacia J2315	YP_002232872.1
Burkholderia cenocepacia J2315	YP_002234481.1
Rhizobium leguminosarum bv. trifolii WSM2304	YP_002282165.1
Aliivibrio salmonicida LFI1238	YP_002265246.1
Acinetobacter baumannii AB0057	YP_002317565.1
Acinetobacter baumannii AB307-0294	YP_002327281.1
Methylocella silvestris BL2	YP_002360442.1
Methylobacterium chloromethanicum CM4	YP_002423669.1
Acidithiobacillus ferrooxidans ATCC 23270	YP_002426405.1
Pseudomonas aeruginosa LESB58	YP_002439140.1
Pseudomonas aeruginosa LESB58	YP_002441565.1
Yersinia pestis CO92	YP_002346031.1
Yersinia pestis CO92	YP_002347420.1
Methylobacterium nodulans ORS 2060	YP_002495630.1
Methylobacterium nodulans ORS 2060	YP_002496260.1
Methylobacterium nodulans ORS 2060	YP_002497058.1
Geobacter sp. FRC-32	YP_002537871.1
Agrobacterium radiobacter K84	YP_002541324.1
Agrobacterium vitis S4	YP_002551489.1
Agrobacterium vitis S4	YP_002549360.1
Sinorhizobium fredii NGR234	YP_002826208.1
Burkholderia glumae BGR1	YP_002909043.1
Candidatus Hamiltonella defensa 5AT (Acyrthosiphon pisum)	YP_002923740.1
Edwardsiella ictaluri 93-146	YP_002934276.1

Variovorax paradoxus S110	YP_002947663.1
Desulfovibrio magneticus RS-1	YP_002955226.1
Methylobacterium extorquens AM1	YP_002965845.1
Methylobacterium extorquens AM1	YP_002966879.1
Rhizobium leguminosarum bv. trifolii WSM1325	YP_002976728.1
Dickeya zeae Ech1591	YP_003002473.1
Pectobacterium carotovorum subsp. carotovorum PC1	YP_003019698.1
Methylobacterium extorquens DM4	YP_003070966.1
Erwinia pyrifoliae Ep1/96	YP_002649215.1
Pectobacterium wasabiae WPP163	YP_003261728.1
Halothiobacillus neapolitanus c2	YP_003262850.1
Edwardsiella tarda EIB202	YP_003296640.1
Dickeya dadantii Ech586	YP_003331715.1
Citrobacter rodentium ICC168	YP_003366470.1
Pantoea ananatis LMG 20103	YP_003520250.1
Erwinia amylovora CFBP1430	YP_003530770.1
Erwinia amylovora ATCC 49946	YP_003538486.1
Sphingobium japonicum UT26S	YP_003546445.1
Shewanella violacea DSS12	YP_003558209.1
Yersinia pestis Z176003	YP_003566926.1
Yersinia pestis Z176003	YP_003568278.1
Rhodobacter capsulatus SB 1003	YP_003576501.1
Burkholderia sp. CCGE1002	YP_003608088.1
Pantoea vagans C9-1	YP_003729883.1
Acinetobacter sp. DR1	YP_003734012.1
Ralstonia solanacearum PSI07	YP_003750860.1
Ralstonia solanacearum PSI07	YP_003749682.1
Ralstonia solanacearum CFBP2957	YP_003744153.1
Erwinia billingiae Eb661	YP_003740503.1
Erwinia billingiae Eb661	YP_003740954.1
Gallionella capsiferriformans ES-2	YP_003847234.1
Dickeya dadantii 3937	YP_003885141.1
Burkholderia sp. CCGE1003	YP_003910269.1
Pantoea vagans C9-1	YP_003930460.1
Ketogulonicigenium vulgare Y25	YP_003964946.1
Enterobacter cloacae SCF1	YP_003941574.1
Rhodomicrobium vannielii ATCC 17100	YP_004012993.1
Asticcacaulis excentricus CB 48	YP_004088230.1
Rhodopseudomonas palustris DX-1	YP_004106681.1
Rhodopseudomonas palustris DX-1	YP_004106954.1
Rhodopseudomonas palustris DX-1	YP_004108425.1

Pantoea sp. At-9b	YP_004115279.1
Mesorhizobium ciceri biovar biserrulae WSM1271	YP_004144716.1
Mesorhizobium ciceri biovar biserrulae WSM1271	YP_004145051.1
Burkholderia sp. CCGE1001	YP_004230809.1

rsaL homolgues

Pseudomonas aeruginosa PAO1	NP_250122.1
Burkholderia xenovorans LB400	YP_554692.1
Pseudomonas aeruginosa UCBPP-PA14	YP_791821.1
Pseudomonas aeruginosa PA7	YP_001349252.1
Burkholderia phymatum STM815	YP_001860598.1
Burkholderia phytofirmans PsJN	YP_001888023.1
Pseudomonas aeruginosa LESB58	YP_002441566.1
Laribacter hongkongensis HLHK9	YP_002794907.1
Burkholderia sp. CCGE1002	YP_003608087.1
Gallionella capsiferriformans ES-2	YP_003847233.1
Burkholderia sp. CCGE1003	YP_003910270.1

rsaM homologues

Burkholderia thailandensis E264	YP_439707.1
Burkholderia ambifaria AMMD	YP_776004.1
Burkholderia pseudomallei 668	YP_001062653.1
Burkholderia vietnamiensis G4	YP_001117675.1

The potential homologues were identified using multiple alignment by the CLUSTAL program (accessed via the Wageningen Webportal, http://www.bioinformatics.nl/tools/clustalw.html), building of HMM recognizers (program package HMMER 3.0, http://hmmer.janelia.org/), and running HMMSEARCH program from the same package on full proteomes or selected protein sequences.

The chromosomal positions were then extracted from the .ptt files of the genomes and gene arrangements containing at least one pair of the four gene types within a distance of 3000 nucleotides were analyzed manually before accepted as a QS circuit and listed in Tables 1-2. As this analysis is not based on experimental validation, the

genes listed here should be considered, at least in principle, as putative homologues of the QS genes. We note that the QS circuits of Pseudomonas species are experimentally validated according to the cited sources.
